# Clinical, Echocardiographic, and Electrocardiographic Predictors of Persistent Atrial Fibrillation after Dual-Chamber Pacemaker Implantation: An Integrated Scoring Model Approach

**DOI:** 10.1371/journal.pone.0160422

**Published:** 2016-08-01

**Authors:** Min Soo Cho, Jun Kim, Ju Hyeon Kim, Minsu Kim, Ji Hyun Lee, You Mi Hwang, Uk Jo, Gi-Byoung Nam, Kee-Joon Choi, You-Ho Kim

**Affiliations:** Heart Institute, University of Ulsan College of Medicine, Asan Medical Center, Seoul, Korea; Providence VA Medical Center and Brown University, UNITED STATES

## Abstract

Persistent atrial fibrillation (PeAF) predictors after dual-chamber pacemaker (PM) implantation remain unclear. We sought to determine these predictors and establish an integrated scoring model. Data were retrospectively reviewed for 649 patients (63.8 ± 12.3 years, 48.6% male, mean CHA_2_DS_2_–VASC score 2.7 ± 2.0) undergoing dual-chamber PM implantation. PeAF was defined as documented AF on two consecutive electrocardiograms acquired ≥7 days apart. During a 7.1-year median follow-up (interquartile range 4.5–10.1 years), 67 (10.3%) patients had PeAF. Multivariable analysis showed the following independent predictors of future PeAF: ischemic stroke or transient ischemic accident history (hazard ratio [HR] 2.03, 95% confidence interval [CI] 1.03–3.50, p = 0.040), atrial fibrillation/flutter history (HR 1.80, 95% CI 1.01–3.20, p = 0.046), sinus node disease (HR 2.24, 95% CI 1.16–4.35, p = 0.016), left atrial enlargement (>45 mm, HR 2.14, 95% CI 1.26–3.63, p = 0.005), and time in automatic mode switching >1% at first follow-up interrogation (HR 2.58, 95% CI 1.51–4.42, p < 0.001). An integrated scoring model combining these predictors showed good discrimination performance at the seven-year follow-up. (C-statistic 0.716, 95% CI 0.629–0.802, p < 0.001). Significantly greater seven-year PeAF incidences were seen in patients with higher scores (2–5) than in those with lower scores (0–1) (22.8% ± 3.8% vs. 5.3% ± 1.7%, p < 0.001). In conclusion, an integrated scoring model combining clinical, echocardiographic, and electrocardiographic characteristics is useful for predicting future PeAF in patients with a dual-chamber PM.

## Introduction

Persistent/permanent atrial fibrillation (PeAF) is a clinically important atrial arrhythmia seen after the implantation of a permanent pacemaker (PM). It has a reported incidence ranging 2%–12% according to various definitions of PeAF [1–6]. Atrial fibrillation (AF) after PM implantation is progressive in nature [2,4,7–9], and some previous studies have reported that prolonged duration of AF after PM is associated with increased risk of mortality, stroke, or systemic embolism [6,10,11]. Indeed, as recently developed advanced pacing modes have been shown to decrease PeAF progression in clinical trials, the importance of defining a group at high risk of PeAF, who would benefit from early application of these recent innovations, has increased [12].

Traditionally, VVI pacing modes, or non-physiological ventricular pacing, which inevitably cause AV dissociation and increase cumulative V pacing, have been known to be associated with future PeAF [1,3,7,13]. However, predictors of PeAF after the implantation of a dual-chamber pacemaker, which has become current practice, remain unclear. Although some previous studies have suggested that predictors of future PeAF include a prior history of atrial arrhythmia [1,3,5,14], sinus node disease (SND) [1], the cumulative percentage of ventricular pacing [14], left atrial enlargement (LAE), or decreased LA function [15,16], most of these studies were limited by short duration of follow-up or limited data confined to the patient’s clinical characteristics and baseline evaluations.

Recently developed advanced pacemaker technologies show excellent diagnostic capabilities and may serve as a new source of data for evaluating the electrophysiological status of cardiac chambers and detecting non-manifested atrial arrhythmia during follow-up [4,11,17,18]. We therefore hypothesized that, in addition to the patient’s baseline characteristics and diagnostic evaluations, data related to the PM may be associated with, and even predict, future PeAF. To test our hypothesis, we comprehensively evaluated the characteristics, evaluations, PM data, and long-term outcomes of patients who received a dual-chamber PM in our institute, and used this to identify independent predictors of PeAF. In addition, we established an integrated scoring model that combines all these factors to predict in practice future PeAF.

## Methods

### Study population

**Fig 1:**
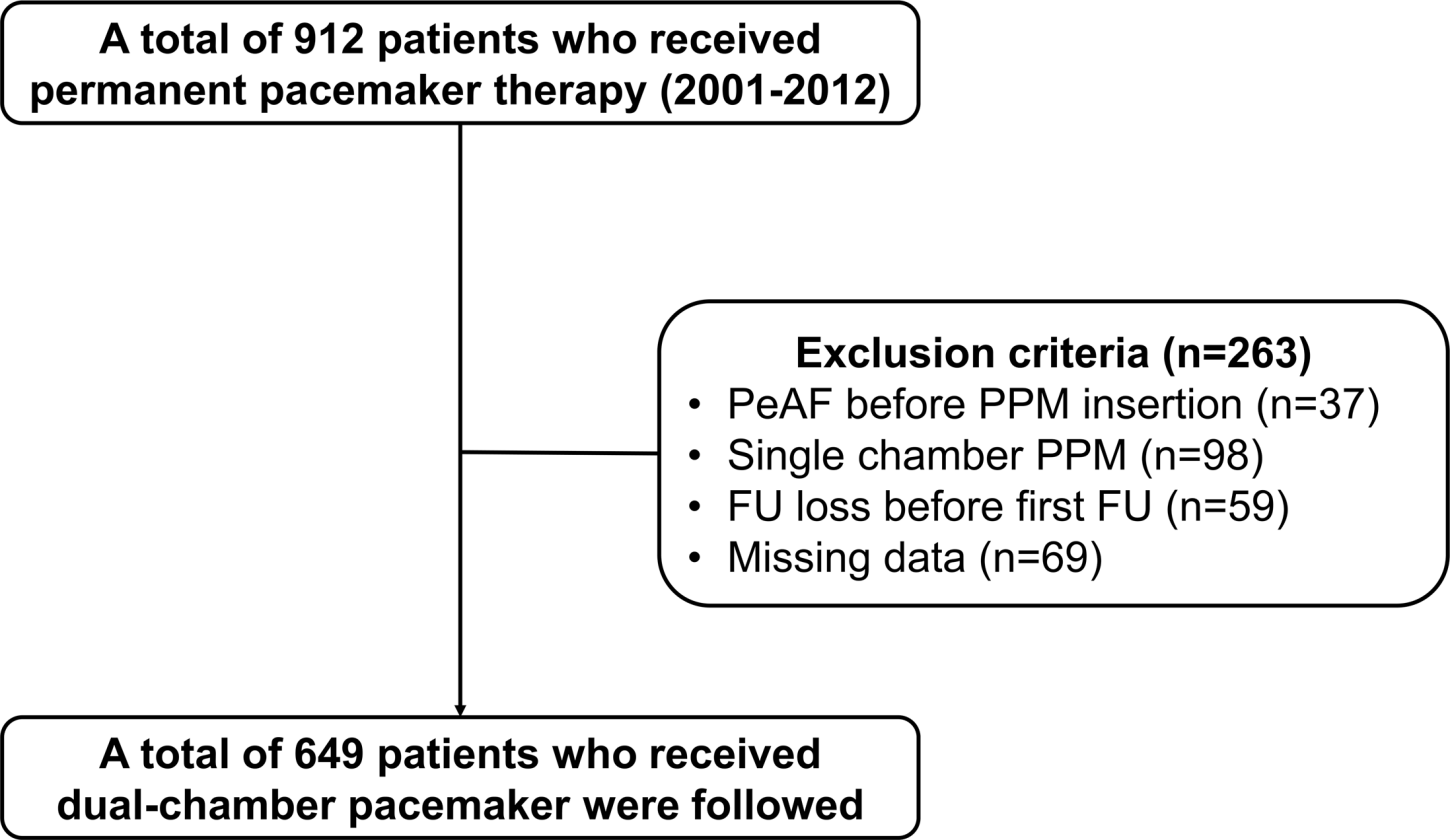
Flow diagram of study patients.

A total of 912 patients who received a PM in the period January 2001 to December 2012 were initially included in the study (Fig 1). After excluding patients with PeAF before PM insertion (n = 37), a single-chamber PM (n = 98), follow-up loss before the first scheduled follow-up (n = 59), or significant missing values (n = 69), data from 649 patients who received dual-chamber pacemakers were evaluated in this analysis. This retrospective study was conducted according to the ethical guidelines of the 1975 Declaration of Helsinki and received *a priori* approval by our Institutional Review Board. The requirement for informed consent was exempted by the board for this study.

### Data collection and follow-up

Clinical, laboratory, and outcome data were obtained via careful review of the medical records of all patients by independent research personnel. Clinical baseline characteristics, comorbidity status, and medications for all patients were acquired from review of medical records at the time of admission for pacemaker insertion. Echocardiographic data from before the index procedure was used in the analysis, and measurements were made according to guidelines from the American Society of Echocardiography [19]. Diagnosis of bradyarrhythmia was confirmed by a review of all available medical records, 12-lead electrocardiograms (ECGs), Holter monitoring, or telemetry data. Diagnosis of SND was made in symptomatic patients with documented sinus pause or block, and tachycardia–bradycardia syndrome.

The pacemakers from all enrolled patients were programmed to the DDD mode at the time of discharge. Patients were initially followed for three months after the index procedure and then once a year afterwards. The follow-up interval was shortened in patients with clinically important arrhythmias, or significant comorbidities. Interrogation data on measured parameters and PM settings were gathered the day after the index procedure and at the first outpatient visit. Data regarding sensed P- or R-wave amplitude, pacing threshold, and impedance of leads were also obtained at after the index procedure and at the first outpatient visit, while data from event counters, including the cumulative percentage of A or V pacing and the numbers of times or percentage of time spent in the automatic mode switching (AMS) mode, were acquired at follow-up visits. The atrial tachycardia detection rate for the triggering of AMS during the initial follow-up period was usually set to 225 beats per minute for St. Jude models (n = 564, 86.9%), and 175 beats per minutes for Medtronic models (n = 78, 12.0%). The activation of AMS during the early follow-up period was decided by the attending physician, and follow-up information was obtained from outpatient visits, telephone contacts with living patients or family members, or review of medical records from other hospitals, as appropriate. The median follow-up duration of the study population was 7.1 years (interquartile range [IQR] 4.5–10.1 years); this is equivalent to 4,475 person-years.

### Study outcome and definitions

The main outcome of this study was to determine PeAF incidence, which was defined as documented AF on two consecutive ECGs acquired ≥7 days apart using 12-lead ECG, Holter monitoring, telemetry, or interrogation data [1,4,20]. AF episodes detected only on device event counters or histograms were not considered as a study outcome unless they were corroborated by other modalities. Differentiation between persistent and permanent AF was also not considered because diagnosis of these was driven by the attending physician’s decision to revert to a sinus rhythm.

### Statistical analysis

Summary statistics are presented as frequencies, percentages, means ± SD, or medians (interquartile range). For continuous variables, the unpaired Student’s t-test and the Mann–Whitney U test were used to assess differences between groups. Chi-square tests and Fisher’s exact test were used to compare frequencies of categorical variables, and receiver operating characteristic (ROC) analysis was used to determine optimal cut-off values and to assess the model’s discrimination performance. Pairwise comparisons between ROC curves were made using Delong’s method, and a Cox proportional hazards model was used to identify factors associated with the development of PeAF. Variables with a p value ≤ 0.1 in univariate analyses, and those with significant clinical relevance, were included in the multivariable Cox regression model. The final models for study outcome were determined using the backward stepwise elimination procedure. The discrimination performance of the model was assessed using the c-statistic, and model calibration was assessed using the Hosmer–Lemeshow test. Because of the limited overall study population and clinical outcomes, the discrimination performance of the model was validated in two ways: (1) using a three-fold split-sample validation procedure, and; (2) using the bootstrap method. In the split-sample procedure, we divided the total sample randomly into three separate groups, and used two-thirds of the population as a training set and the remaining one-third as a test set. The area under the ROC curve (AUC) for each dataset was calculated. To improve robustness, this procedure was repeated three times for each dataset used as either a training test or a test set. The bootstrap method involved acquiring 1,000 random samples from the original dataset and then estimating the distribution of AUC values. Cumulative survival, and event-free survival, rate curves were generated using the Kaplan–Meier method and compared using the log-rank test. All p values were two-sided, and a value of p < 0.05 was considered significant; statistical analyses were performed using SPSS (version 18.0; SPSS Inc., Chicago, IL) and R software version 3.1.2.

## Results

### Baseline characteristics

PeAF was diagnosed in 67 (10.3%) patients during the follow-up period. The baseline characteristics of the patients with, and without, PeAF are summarized in Table 1; mean age (64.5 ± 9.5 vs. 63.9 ± 12.6, p = 0.773), proportion of male patients (46.6% vs. 48.8%, p = 0.695), mean CHA_2_DS_2_–VASC score (3.0 ± 1.8 vs. 2.7 ± 1.7, p = 0.153), and CHADS_2_ score (1.7 ± 1.4 vs. 1.5 ± 1.3, p = 0.223) did not differ significantly between patients with, and without, PeAF. History of previous stroke or transient ischemic accident (stroke/TIA, 22.4% vs. 11.2%, p = 0.008), and AF or atrial flutter (AF/AFL, 52.2% vs. 14.6%, p < 0.001), were more frequent in the patients with PeAF than in those without. Using baseline echocardiography, left atrial size was shown to be significantly larger (45.0 ± 8.4 vs. 40.9 ± 7.0 mm, p < 0.001), and LA enlargement (LAE), defined as a LA of more than 45 mm, was more frequently found in the PeAF group (47.8% vs. 27.3%, p < 0.001). The number of patients with mitral stenosis or a prosthetic heart valve was not significantly different between the groups, whereas moderate, or severe, tricuspid regurgitation (TR) (19.4% vs. 7.1%, p < 0.001) and SND as an indication of PM (70.1% vs. 32.6%, p < 0.001) were more frequent in the PeAF group. In terms of discharge medication, anti-arrhythmic drugs were more frequently prescribed in the PeAF group, reflecting a higher prevalence of prior AF or AFL in this group.

**Table 1:** Baseline characteristics of the study population.

Variables	No PeAF (n = 582)	PeAF (n = 67)	p
Age	63.9 ± 12.6	64.5 ± 9.5	0.773
Male, n (%)	284 (48.8)	31 (46.6)	0.695
Body mass index	23.9 ± 3.3	24.6 ± 2.8	0.067
Hypertension, n (%)	385 (66.2)	49 (73.1)	0.250
Diabetes mellitus, n (%)	151 (25.9)	15 (22.4)	0.527
Peripheral arterial disease, n (%)	15 (2.6)	1 (1.5)	0.588
Prior Stroke/TIA, n (%)	65 (11.2)	15 (22.4)	0.008
Coronary artery disease, n (%)	136 (23.4)	21 (31.3)	0.149
Valvular heart disease, n (%)	76 (13.1)	13 (19.4)	0.153
Congestive heart failure, n (%)	85 (14.6)	9(13.4)	0.796
COPD, n (%)	28 (4.8)	4 (6.0)	0.678
Chronic renal failure, n (%)	51 (8.8)	6 (9.0)	0.958
Prior cardiac surgery, n (%)	89 (15.3)	13 (19.4)	0.381
Prior AF/AFL, n (%)	85 (14.6)	35 (52.2)	0.001
Mean CHA_2_DS_2_–VASC score	2.7 ± 1.7	3.0 ± 1.8	0.153
CHA_2_DS_2_–VASC score distribution			0.207
0	43 (7.4)	6 (9.0)	
1	112 (19.2)	7 (10.4)	
≥2	427 (73.4)	54 (80.6)	
Mean CHADS_2_ score	1.5 ± 1.3	1.7 ± 1.4	0.223
CHADS_2_ score distribution			0.731
0	135 (23.2)	13 (19.4)	
1	202 (34.7)	23 (34.3)	
≥2	245 (42.1)	31 (46.3)	
**Echocardiography data**			
LA, mm	40.9 ± 7.0	45.0 ± 8.4	0.001
LA > 45 mm, n (%)	159 (27.3)	32 (47.8)	0.001
LVEF, %	59.4 ± 9.6	57.7 ± 9.7	0.153
RV–RA PG, mmHg	26.9 ± 10.3	26.3 ± 8.0	0.597
Moderate TR, n (%)	41 (7.1)	13 (19.4)	0.001
Mitral stenosis, or prosthetic heart valves, n (%)	26 (4.5)	6 (6.0)	0.579
**ECG data**			
Sinus node disease, n (%)	190 (32.6)	47 (70.1)	0.001
Heart rate	48.9 ± 14.2	53.3 ± 14.5	0.018
QRS width	108.7 ± 28.0	103.9 ± 22.3	0.178
**Discharge medication**			
Antiarrhythmic drugs, n (%)	33 (5.7)	14 (20.9)	0.001
Beta-blockers, n (%)	71 (12.2)	9 (13.4)	0.771
Non-DPH CCB, n (%)	33 (5.7)	5 (7.5)	0.554
Digoxin, n (%)	13 (2.2)	3 (4.5)	0.262

Data are presented as mean ± SD, median (interquartile range), or number (%). Abbreviations: TIA, transient ischemic accident; COPD, chronic obstructive pulmonary disease; AF, atrial fibrillation; AFL, atrial flutter; LA, left atrium; LVEF, left ventricular ejection fraction; RV, right ventricle; RA, right atrium; PG, pressure gradient; TR, tricuspid regurgitation; DPH, diphenhydramine; CCB, calcium channel blocker.

Regarding the immediate post-procedure PM interrogation data acquired the day after index procedure, sensed signal amplitude, pacing threshold, and impedance of both chambers did not differ significantly between the groups (Table 2). However, in the first follow-up interrogation, acquired a median of 88 days (IQR 33–126) after discharge, measured P-wave amplitude was lower (median 2.0 mV [IQR 1.4–2.9] vs. 2.7 mV [IQR 1.8–4.0], p = 0.001), and the proportion of patients with a low P-wave amplitude (≤2.5 mV), defined as being below the median value for the total population, was significantly higher in the PeAF group than in the non-PeAF group (67.2% vs. 49.0%, p = 0.005). The percentage of cumulative atrial pacing was significantly higher in the PeAF group (46.9% ± 32.2% vs. 31.2% ± 33.7%, p < 0.001), which may reflect the higher SND incidence in these patients. Among the subset of patients for whom AMS was activated during the initial follow-up period (n = 431, 66.4% of the total population), the percentage with an AMS event was greater in the PeAF group than in the non-PeAF group (76.7% [46 of 60] vs. 44.5% [165 of 371]), p < 0.001), as was the percentage of time spent in the AMS-activated state (AMS burden, 10.4v ± 19.7% vs. 2.0% ± 9.3, p = 0.017).

**Table 2:** Pacemaker settings and data acquired immediately post-procedure and at follow-up interrogation.

Variables	No PeAF (n = 582)	PeAF (n = 67)	p
**Immediately post-procedure**			
Measured P-wave amplitude, mV	2.8 (1.8–3.6)	2.5 (1.8–0.5)	0.477
A pacing threshold, mV	0.6 ± 0.2	0.7 ± 0.4	0.024
A lead impedance, ohm	478.0 ± 100.8	453.0 ± 78.9	0.050
Measured R-wave amplitude, mV	10.3 (7.9–12.5)	9 (7–12.5)	0.238
V pacing threshold, mV	0.6 ± 0.2	0.6 ± 0.2	0.072
V lead impedance, ohm	618.8 ± 124.4	596.3 ± 118.8	0.161
SAVI	152.2 ± 21.4	159.3 ± 26.7	0.041
PAVI	172.8 ± 20.3	178.5 ± 24.6	0.074
Follow-up interrogation*			
Measured P-wave amplitude, mV,	2.7 (1.8–4)	2.0 (1.4–2.9)	0.001
Low P amplitude, n (%) †	285 (49.0)	45 (67.2)	0.005
A pacing threshold, mV	0.8 ± 0.4	0.8 ± 0.3	0.151
A lead impedance, ohm	473.1 ± 85.8	460.3 ± 78.7	0.248
Measured R-wave amplitude, mV,	11 (8–12.5)	11 (8–12.5)	0.949
Measured R amplitude, mV	10.6 ± 4.4	10.3 ± 3.2	0.566
V pacing threshold, mV	1.0 ± 0.3	1.0 ± 0.4	0.899
V lead impedance, ohm	579.4 ± 113.5	559.7 ± 118.5	0.182
Cumulative A pacing, %	31.2 ± 33.7	46.9 ± 32.2	0.001
Cumulative V pacing, %	75.2 ± 36.2	67.7 ± 34.6	0.105
**AMS-activated patients**	**n = 371**	**n = 60**	
Patients with events, n (%)	165 (44.5)	46 (76.7)	0.001
Burden, %	2.0 ± 9.3	10.4 ± 19.7	0.017
Burden > 1%, n (%)	52 (14.0)	28 (46.7)	0.001

Data are presented as mean ± SD, median (interquartile range), or number (%). Abbreviations: A, atrium; V, ventricle; SAVI, sensed atrioventricular interval; PAVI, paced atrioventricular interval; AMS, automatic mode switching.

*Data acquired at a median of 88 days (interquartile range 33–126) after the index procedure

†Sensed P-wave amplitude ≤ 2.5 mV.

### Predictors of persistent atrial fibrillation

The predictive value of each variable was analyzed using a Cox proportional hazards model (Table 3). In univariable analysis, prior stroke/TIA, prior AF/AFL, SND, LAE, moderate or severe TR, low P-wave amplitude at the first follow-up (≤2.5 mV), and cumulative atrial pacing were significantly associated with future PeAF. In the subset of AMS-activated patients (n = 431), in addition to the aforementioned variables, high AMS burden (>1%) was also associated with PeAF.

**Table 3:** Predictors of persistent or permanent atrial fibrillation.

	Univariable analysis		Multivariable analysis		Multivariable model on AMS-activated patients (n = 431)	
Variable	HR (95% CI)	p	HR (95% CI)	p	HR (95% CI)	p
Age (per single year)	1.02 (0.99–1.04)	0.153				
Male	0.74 (0.48–1.20)	0.226				
BMI	1.05 (0.98–1.13)	0.175				
Prior Stroke/TIA	2.55 (1.43–4.54)	0.001	2.07 (1.15–3.74)	0.015	2.03 (1.09–3.29)	0.026
Prior AF/AFL	5.40 (3.34–8.72)	0.001	2.84 (1.65–4.89)	< 0.001	1.80 (1.01–3.20)	0.046
SND	4.34 (2.57–7.33)	0.001	2.64 (1.47–4.74)	0.001	2.24 (1.16–4.35)	0.016
LA > 45 mm	2.67 (1.65–4.32)	0.001	2.08 (1.27–3.40)	0.003	2.14 (1.26–3.63)	0.005
Moderate TR	2.74 (1.49–5.03)	0.001				
Low P-wave amplitude at follow-up (≤2.5 mV)	2.21 (1.35–3.61)	0.002				
A pacing (each 1%)	1.01 (1.00–1.02)	0.001				
V pacing (each 1%)	0.99 (0.99–1.00)	0.124				
AMS burden > 1%	3.76 (2.26–6.27)	0.001			2.58 (1.51–4.42)	0.001

Abbreviations: AMS, automatic mode switching; BMI, body mass index; TIA, transient ischemic accident; AF, atrial fibrillation; AFL, atrial flutter; SND; sinus node disease; LA, left atrium; TR, tricuspid regurgitation; A, atrial; V, ventricular.

To identify independent predictors of PeAF, we established two multivariable Cox proportional hazards models according to patient group. Model 1 was developed for the entire study population (n = 649), whereas Model 2 was established for just AMS-activated patients (n = 431). In Model 1, using backward elimination, prior stroke/TIA (hazard ratio [HR] 2.07, 95% confidence interval [CI] 1.15–3.74, p = 0.015), prior AF/AFL (HR 2.84, 95% CI 1.65–4.89, p < 0.001), SND (HR 2.64, 95% CI 1.47–4.74, p = 0.001), and LAE (HR 2.08, 95% CI 1.27–3.40, p = 0.003) were found to be independent predictors of future PeAF for the entire population. In Model 2 (AMS-activated patients, n = 431), prior stoke/TIA (HR 2.03, 95% CI 1.09–3.29, p = 0.026), prior AF/AFL (HR 1.80, 95% CI 1.01–3.20, p = 0.046), SND (HR 2.24, 95% CI 1.16–4.35, p = 0.016), LAE (HR 2.14, 95% CI 1.26–3.63, p = 0.005), and AMS burden > 1% (HR 2.58, 95% CI 1.51–4.42, p < 0.001) were found to be independent predictors of future PeAF.

### Integrated scoring model

**Fig 2:**
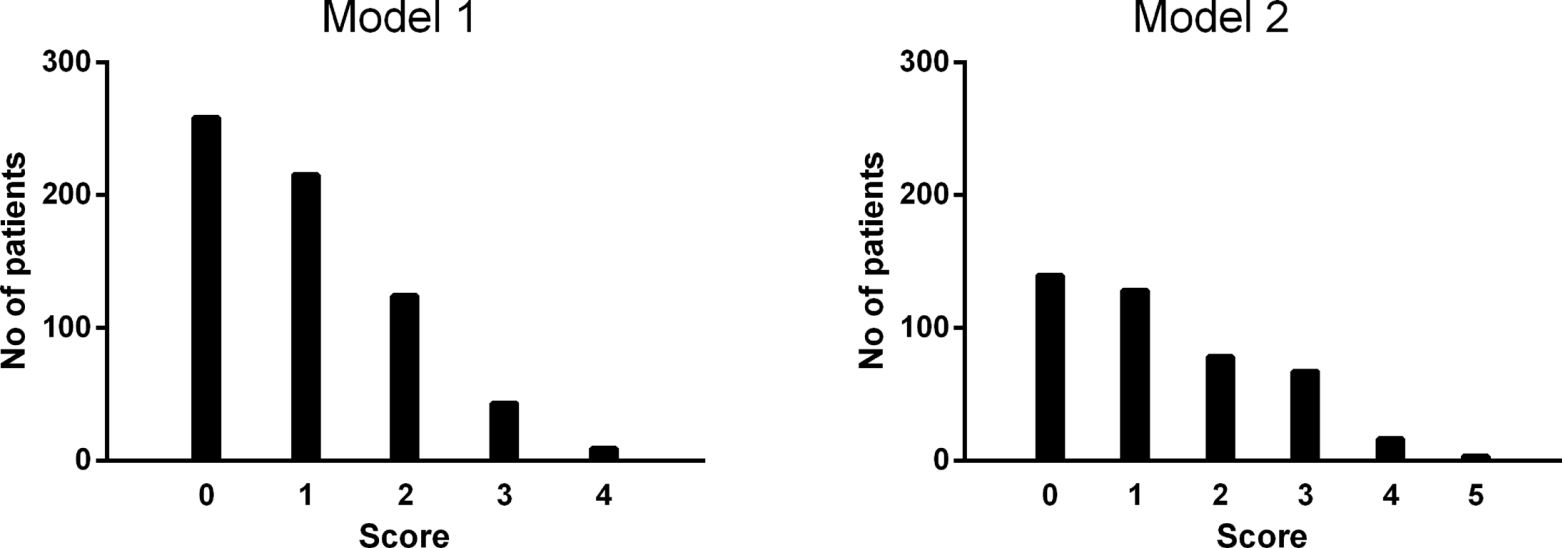
Numbers of patients in models one and two based on integrated scores.

We defined an integrated risk score for each model as the number of independent risk factors present. Thus, the maximum possible scores were four in Model 1 (total population) and five in Model 2 (the AMS-activated patients). Fewer patients were seen in the higher score category (Fig 2), and the discrimination performance of each scoring model after seven years of follow-up are summarized in Table 4. Model 1 showed good discrimination in terms of predicting future PeAF, with a c-statistic of 0.768 (95% confidence interval [CI] 0.690–0.845), a significant improvement (p < 0.001) compared to the previously reported HATCH score system [21]. The sensitivity and specificity of Model 1 at its optimal cutoff value (risk score ≥2) were 72.4% and 76.5%, respectively. In the subset of AMS-activated patients (n = 431), Model 2 also showed significantly better discrimination performance (c-statistic 0.716, 95% CI 0.629–0.802, sensitivity 71.8%, and specificity 66.6% for risk score ≥2) compared with the HATCH score (p < 0.001). For this subset of patients, the c-statistic of Model 2 was slightly better than that of Model 1 in a pairwise comparison (p = 0.025). The good discrimination performance of both models was also robust on the basis of the validation results using both split-sample and bootstrap methods (S1 Table).

**Table 4:** Predictive function of integrated scoring models compared to the HATCH scoring model after seven years of follow-up.

	SN at maximal accuracy (%)†	SP at maximal accuracy (%)†	c-statistic (95% confidence interval)	Hosmer-Lemeshow;—p-value
Total population (n = 649)				
HATCH	47.9	62.6	0.596 (0.508–0.683)	0.860
Model 1	72.4	76.5	0.768 (0.690–0.845)	0.425
AMS-activated population (n = 431)				
HATCH	50.5	67.4	0.609 (0.518–0.701)	0.995
Model 1	68.5	69.9	0.697 (0.608–0.785)	0.688
Model 2	71.8	66.6	0.716 (0.629–0.802)	0.976

† Optimal cut-off value for all three models (HATCH, Model 1, and Model 2) was risk score ≥2. Abbreviations: SN, sensitivity; SP, specificity; AMS, automatic mode switching.

**Fig 3:**
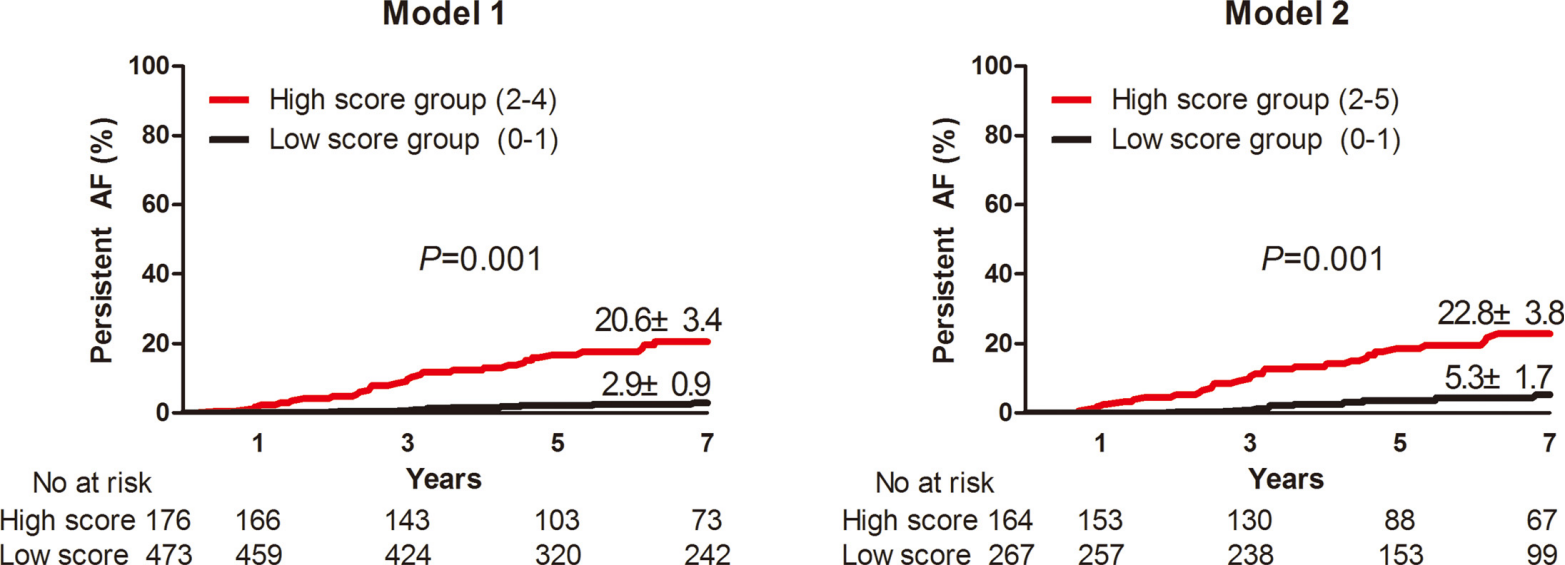
PeAF incidence in low or high-scoring groups defined by integrated scoring models one and two. PeAF incidence in the high-scoring group was significantly greater than in the low-scoring group for both models one (20.6% ± 3.4% vs. 2.9% ± 0.9%, p < 0.001) and two (22.8% ± 3.8% vs. 5.3% ± 1.7%, p < 0.001).

We defined those patients with a score of zero to one as the low-risk group, and those with a score of more than one as high-risk for future PeAF. Using this cut-off, 27.1% of patients (n = 176) in Model 1 and 33.3% (n = 164) in Model 2 were defined as high risk (Fig 2). In the Kaplan–Meier analysis, the high score group showed a significantly higher PeAF incidence after seven years of follow-up in both models one (20.6% ± 3.4% vs. 2.9% ± 0.9%, p < 0.001) and two (22.8% ± 3.8% vs. 5.3% ± 1.7%, p < 0.001, Fig 3).

## Discussion

In this retrospective review of our PM patients, we have made several important findings regarding PeAF after PM. First, documented PeAF incidence after PM was 10.3% after more than seven years follow-up. Second, several independent predictors of future PeAF were identified by a comprehensive review of clinical, echocardiographic, electrocardiographic, and interrogation data. Third, an integrated scoring model that combined these predictors showed excellent discrimination performance, and the addition of interrogation data in the model enhanced this function. Fourth, the high-risk group, defined by this integrated model, showed a significantly higher PeAF incidence during long-term follow-up.

The cited PeAF incidence after PM from previous studies varies greatly between 2% and 12%; this variability may stem from the use of different definitions of PeAF, from clinically diagnosed chronic AF to a more stringent definition of persistent AF based on continuous Holter monitoring [1–6]. The definition of PeAF used in our study (i.e., at least two consecutive positive ECGs acquired ≥7 days apart) was based on reports by Skanes et al. [1], and Sweeny et al. [4]. As our study incorporated only objectively documented PeAF, and all patients were regularly followed up in a single center, our findings provide reliable epidemiological data on PeAF incidence after PM insertion. In addition, considering that the progression of AF is time dependent, and usually involves a long delay [22,23], more than seven years of follow-up with a relatively large number of patients is another major strength of our current study.

Most of the independent predictors identified in our study, including prior AF/AFL, SND, and LAE, have already been evaluated in previous work [1,3,5,14,15,16]. In addition, a firm association between subclinical AF and prior stroke/TIA was established by a recent large trial [11]. As well as these “traditional” risk factors (Model 1), the addition in the present study of PM interrogation data during early follow-up, especially of AMS data, established an even stronger predictive model with excellent discrimination performance (Model 2). Thus, although we do not believe that episodes of AMS activation should be interpreted as episodes of AF/AFL, because triggering of AMS activation is strongly correlated with AF episodes [24,25], we do believe that the cumulative burden of AMS activation during early follow-up may be a surrogate marker for the burden of AF in these patients. Indeed, currently evolving AF-detection algorithms for PMs may potentially provide even better data for diagnosing and predicting future PeAF.

One interesting finding of our study is the association in the univariable analysis between future PeAF and low P-wave amplitude at follow-up, although this was not found in the immediate post-procedure period. Although not demonstrated in previous studies, we believe that low P-wave amplitude at follow-up may reflect electrical remodeling of the atrium, which could be a substrate for future PeAF. Indeed, the work of Brandt et al. showed that low atrial amplitude was associated with old age or SND [26], whereas another study by Sanders et al. showed low atrial voltage in patients with SND and attributed the association between low atrial voltage, SND, and AF to atrial remodeling [27]. In addition to the importance of the P-wave amplitude itself, our data highlights the temporal change in the P-wave after PM, and the importance of serial follow-up of interrogation data. In terms of acquisition timing, we believe that follow-up interrogation data may more precisely reflect the electrophysiological substrate of the cardiac chamber because it would not be affected by inflammation or tissue edema at the time of lead insertion. Although it was not an independent predictor in our multivariable analysis model, there may be an association between low P-wave amplitude and atrial remodeling. Further data is required to verify this hypothesis.

The integrated scoring model in our study incorporated traditional risk factors, and newly identified variables from follow-up interrogation data. Through adequate dichotomization of variables according to their clinical relevance, we developed a scoring model that is both easy to apply and interpret. This model comprehensively combines all available data and may more accurately reflect the patient’s risk than the previous HATCH scoring model, which is mainly dependent on clinical variables [21]. We believe that the high scoring group in our cohort represents a genuine high-risk group that will exhibit a higher PeAF incidence, although further prospective studies evaluating the benefit of the early application of novel AF-preventive pacing methods in these high-risk patients are required [12].

There were several limitations to our study. The inherent limitation of selection bias stemming from the retrospective nature of the analysis was inevitable, and diagnosis of subclinical PeAF could be underestimated as only objectively “documented” cases were defined as a study outcome. In addition, data regarding AF events, or duration, according to the AF-detection algorithms of the PMs were not incorporated into the scoring model, as they only became available in recent years. Similarly, intracardiac electrograms during AMS events have only been available in recent years. Thus, as PeAF after PM develops over a long time period, more data are needed to clarify the value of these algorithms and the use of intracardiac electrograms during AMS events. In addition, the clinical impact of PeAF after PM could not be evaluated because of the limited numbers of patients and short follow-up period, whereas the relatively lower discrimination performance of the HATCH model in our population compared to the original could stem from differences in comorbidities. Finally, as the risk score in our study was not validated in other populations, application of our findings to other patient groups should be treated with caution.

### Conclusion

An integrated scoring model combining clinical, echocardiographic, and electrocardiographic characteristics is useful for predicting future PeAF in patients with dual-chamber PM.

## Supporting Information

S1 Table Area under the receiver operating characteristic curve for the training and testing the data set for scoring Models 1 and 2. (DOCX)

## Abbreviations

A: atrial
AF: atrial fibrillation
AFL: atrial flutter
AMS: automatic mode switching
ECG: electrocardiogram
LAE: left atrial enlargement
PeAF: persistent/permanent atrial fibrillation
PM: pacemaker
SND: sinus node disease
TIA: transient ischemic attack
V: ventricular

## References

[1] Skanes AC, Krahn AD, Yee R, Klein GJ, Connolly SJ, Kerr CR, et al Progression to chronic atrial fibrillation after pacing: the Canadian Trial of Physiologic Pacing. CTOPP Investigators. Journal of the American College of Cardiology. 2001;38:167–72. 11451268 10.1016/s0735-1097(01)01326-2

[2] Gillis AM, Morck M. Atrial fibrillation after DDDR pacemaker implantation. Journal of cardiovascular electrophysiology. 2002;13:542–7. 12108493 10.1046/j.1540-8167.2002.00542.x

[3] Stambler BS, Ellenbogen KA, Orav EJ, Sgarbossa EB, Estes NA, Rizo-Patron C, et al Predictors and clinical impact of atrial fibrillation after pacemaker implantation in elderly patients treated with dual chamber versus ventricular pacing. Pacing and clinical electrophysiology: PACE. 2003;26:2000–7. 14516342 10.1046/j.1460-9592.2003.00309.x

[4] Sweeney MO, Bank AJ, Nsah E, Koullick M, Zeng QC, Hettrick D, et al Minimizing ventricular pacing to reduce atrial fibrillation in sinus-node disease. The New England journal of medicine. 2007;357:1000–8. 17804844 10.1056/NEJMoa071880

[5] Padeletti L, Muto C, Maounis T, Schuchert A, Bongiorni MG, Frank R, et al Atrial fibrillation in recipients of cardiac resynchronization therapy device: 1-year results of the randomized MASCOT trial. American heart journal. 2008;156:520–6. 10.1016/j.ahj.2008.04.013 18760135

[6] Petrac D, Radeljic V, Delic-Brkljacic D, Manola S, Cindric-Bogdan G, Pavlovic N. Persistent atrial fibrillation is associated with a poor prognosis in patients with atrioventricular block and dual-chamber pacemaker. Pacing and clinical electrophysiology: PACE. 2012;35:695–702. 10.1111/j.1540-8159.2012.03376.x 22452373

[7] Mattioli AV, Vivoli D, Mattioli G. Influence of pacing modalities on the incidence of atrial fibrillation in patients without prior atrial fibrillation. A prospective study. European heart journal. 1998;19:282–6. 9519322 10.1053/euhj.1997.0616

[8] Saksena S, Hettrick DA, Koehler JL, Grammatico A, Padeletti L. Progression of paroxysmal atrial fibrillation to persistent atrial fibrillation in patients with bradyarrhythmias. American heart journal. 2007;154:884–92. 17967594 10.1016/j.ahj.2007.06.045

[9] Nagarakanti R, Saksena S, Hettrick D, Koehler JL, Grammatico A, Padeletti L. Progression of new onset to established persistent atrial fibrillation: an implantable device-based analysis with implications for clinical classification of persistent atrial fibrillation. Journal of interventional cardiac electrophysiology: an international journal of arrhythmias and pacing. 2011;32:7–15. 21766221 10.1007/s10840-011-9601-1

[10] Capucci A, Santini M, Padeletti L, Gulizia M, Botto G, Boriani G, et al Monitored atrial fibrillation duration predicts arterial embolic events in patients suffering from bradycardia and atrial fibrillation implanted with antitachycardia pacemakers. Journal of the American College of Cardiology. 2005;46:1913–20. 16286180 10.1016/j.jacc.2005.07.044

[11] Healey JS, Connolly SJ, Gold MR, Israel CW, Van Gelder IC, Capucci A, et al Subclinical atrial fibrillation and the risk of stroke. New England Journal of Medicine. 2012;366:120–9. 10.1056/NEJMoa1105575 22236222

[12] Boriani G, Tukkie R, Manolis AS, Mont L, Purerfellner H, Santini M, et al Atrial antitachycardia pacing and managed ventricular pacing in bradycardia patients with paroxysmal or persistent atrial tachyarrhythmias: the MINERVA randomized multicentre international trial. European heart journal. 2014;35:2352–62. 10.1093/eurheartj/ehu165 24771721 PMC4163193

[13] Kerr CR, Connolly SJ, Abdollah H, Roberts RS, Gent M, Yusuf S, et al Canadian Trial of Physiological Pacing: Effects of physiological pacing during long-term follow-up. Circulation. 2004;109:357–62. 14707022 10.1161/01.CIR.0000109490.72104.EE

[14] Ricci RP, Botto GL, Benezet JM, Nielsen JC, Roy LD, Piot O, et al Association between ventricular pacing and persistent atrial fibrillation in patients indicated to elective pacemaker replacement: Results of the Prefer for Elective Replacement MVP (PreFER MVP) randomized study. Heart rhythm: the official journal of the Heart Rhythm Society. 2015;12:2239–46. 10.1016/j.hrthm.2015.06.041 26142300

[15] Healey JS, Martin JL, Duncan A, Connolly SJ, Ha AH, Morillo CA, et al Pacemaker-detected atrial fibrillation in patients with pacemakers: prevalence, predictors, and current use of oral anticoagulation. The Canadian journal of cardiology. 2013;29:224–8. 10.1016/j.cjca.2012.08.019 23142343

[16] Kosmala W, Saito M, Kaye G, Negishi K, Linker N, Gammage M, et al Incremental value of left atrial structural and functional characteristics for prediction of atrial fibrillation in patients receiving cardiac pacing. Circulation. Cardiovascular imaging. 2015;8 10.1161/CIRCIMAGING.114.002942 25862716

[17] Glotzer TV, Hellkamp AS, Zimmerman J, Sweeney MO, Yee R, Marinchak R, et al Atrial high rate episodes detected by pacemaker diagnostics predict death and stroke: report of the Atrial Diagnostics Ancillary Study of the MOde Selection Trial (MOST). Circulation. 2003;107:1614–9. 12668495 10.1161/01.CIR.0000057981.70380.45

[18] Orlov MV, Ghali JK, Araghi-Niknam M, Sherfesee L, Sahr D, Hettrick DA, et al Asymptomatic atrial fibrillation in pacemaker recipients: incidence, progression, and determinants based on the atrial high rate trial. Pacing and clinical electrophysiology: PACE. 2007;30:404–11. 17367361 10.1111/j.1540-8159.2007.00682.x

[19] Lang RM, Bierig M, Devereux RB, Flachskampf FA, Foster E, Pellikka PA, et al Recommendations for chamber quantification: a report from the American Society of Echocardiography's Guidelines and Standards Committee and the Chamber Quantification Writing Group, developed in conjunction with the European Association of Echocardiography, a branch of the European Society of Cardiology. Journal of the American Society of Echocardiography: official publication of the American Society of Echocardiography. 2005;18:1440–63. 16376782 10.1016/j.echo.2005.10.005

[20] January CT, Wann LS, Alpert JS, Calkins H, Cigarroa JE, Cleveland JC Jr., et al 2014 AHA/ACC/HRS guideline for the management of patients with atrial fibrillation: a report of the American College of Cardiology/American Heart Association Task Force on Practice Guidelines and the Heart Rhythm Society. Journal of the American College of Cardiology. 2014;64:e1–76. 10.1016/j.jacc.2014.03.022 24685669

[21] de Vos CB, Pisters R, Nieuwlaat R, Prins MH, Tieleman RG, Coelen RJ, et al Progression from paroxysmal to persistent atrial fibrillation clinical correlates and prognosis. Journal of the American College of Cardiology. 2010;55:725–31. 10.1016/j.jacc.2009.11.040 20170808

[22] Al-Khatib SM, Wilkinson WE, Sanders LL, McCarthy EA, Pritchett EL. Observations on the transition from intermittent to permanent atrial fibrillation. American heart journal. 2000;140:142–5. 10874276 10.1067/mhj.2000.107547

[23] Kerr CR, Humphries KH, Talajic M, Klein GJ, Connolly SJ, Green M, et al Progression to chronic atrial fibrillation after the initial diagnosis of paroxysmal atrial fibrillation: results from the Canadian Registry of Atrial Fibrillation. American heart journal. 2005;149:489–96. 15864238 10.1016/j.ahj.2004.09.053

[24] Passman RS, Weinberg KM, Freher M, Denes P, Schaechter A, Goldberger JJ, et al Accuracy of mode switch algorithms for detection of atrial tachyarrhythmias. Journal of cardiovascular electrophysiology. 2004;15:773–7. 15250860 10.1046/j.1540-8167.2004.03537.x

[25] de Voogt WG, van Hemel NM, van de Bos AA, Koistinen J, Fast JH. Verification of pacemaker automatic mode switching for the detection of atrial fibrillation and atrial tachycardia with Holter recording. Europace: European pacing, arrhythmias, and cardiac electrophysiology: journal of the working groups on cardiac pacing, arrhythmias, and cardiac cellular electrophysiology of the European Society of Cardiology. 2006;8:950–61. 10.1093/europace/eul112 17043069

[26] Brandt J, Attewell R, FÅHraeus T, SchÜLler H. Acute Atrial Endocardial P Wave Amplitude and Chronic Pacemaker Sensitivity Requirements: Relation to Patient Age and Presence of Sinus Node Disease. Pacing and Clinical Electrophysiology. 1990;13:417–24. 1692125 10.1111/j.1540-8159.1990.tb02056.x

[27] Sanders P, Morton JB, Kistler PM, Spence SJ, Davidson NC, Hussin A, et al Electrophysiological and electroanatomic characterization of the atria in sinus node disease: evidence of diffuse atrial remodeling. Circulation. 2004;109:1514–22 15007004 10.1161/01.CIR.0000121734.47409.AA

