# Supplementary material for: Clinical, Echocardiographic, and Electrocardiographic Predictors of Persistent Atrial Fibrillation after Dual-Chamber Pacemaker Implantation: An Integrated Scoring Model Approach
Source: PLoS One. 2016 Aug 1;11(8):e0160422. doi: 10.1371/journal.pone.0160422 (PMC4968832; doi:10.1371/journal.pone.0160422)
Supplement: S1 Table — (DOCX) [file pone.0160422.s001.docx]

**Supplementary Table 1**. Area under the receiver operating characteristic curve for the training and testing the data set for scoring Models 1 and 2.

|  | Model 1 | | Model 2 | |
| --- | --- | --- | --- | --- |
| Dataset | HR | 95% CI | HR | 95% CI |
| Training Set 1 | 0.783 | 0.707–0.858 | 0.830 | 0.730–0.876 |
| Testing Set 1 | 0.741 | 0.630–0.852 | 0.786 | 0.677–0.896 |
| Training Set 2 | 0.749 | 0.669–0.829 | 0.786 | 0.712–0.860 |
| Testing Set 2 | 0.810 | 0.715–0.906 | 0.825 | 0.719–0.931 |
| Training Set 3 | 0.776 | 0.703–0.850 | 0.805 | 0.729–0.881 |
| Testing Set 3 | 0.755 | 0.639–0.871 | 0.785 | 0.683–0.887 |
| Bootstrapped Set  (1000 replications) | 0.770 | 0.700–0.831 | 0.796 | 0.730–0.853 |
